# The Detrimental Impact of Maladaptive Personality on Public Mental Health: A Challenge for Psychiatric Practice

**DOI:** 10.3389/fpsyt.2015.00087

**Published:** 2015-06-09

**Authors:** Michael Pascal Hengartner

**Affiliations:** ^1^Department of Applied Psychology, Zurich University of Applied Sciences (ZHAW), Zurich, Switzerland

**Keywords:** review, personality, epidemiology, psychopathology, psychiatric practice, public health, personality disorders, nosology

## Abstract

Experts in personality psychology and personality disorders have long emphasized the pervasive and persistent detrimental impact of maladaptive personality traits on mental health and functioning. However, in routine psychiatric practice, maladaptive personality is readily ignored and personality traits are seldom incorporated into clinical guidelines. The aim of this narrative review is to outline how pervasively personality influences public mental health and how personality thereby challenges common psychiatric practice. A comprehensive search and synthesis of the scientific literature demonstrates that maladaptive personality traits and personality disorders, in particular high neuroticism and negative affectivity, first, are risk factors for divorce, unemployment, and disability pensioning; second, relate to the prevalence, incidence, and co-occurrence of common mental disorders; third, impair functioning, symptom remission, and recovery in co-occurring common mental disorders; and fourth, predispose to treatment resistance, non-response and poor treatment outcome. In conclusion, maladaptive personality is not only involved in the development and course of mental disorders but also predisposes to chronicity and re-occurrence of psychopathology and reduces the efficacy of psychiatric treatments. The pernicious impact of maladaptive personality on mental health and functioning demands that careful assessment and thorough consideration of personality should be compulsory in psychiatric practice.

## Introduction

Recently, a special series published in the Lancet (1) drew the mental health profession’s attention to the frequently ignored diagnosis of personality disorders (PDs). In the introduction to their paper, Tyrer et al. (1) stress the relevance of PDs for both mental health policy makers and medical practitioners, and legitimately warn that this highly impairing and burdensome condition is too often overlooked in clinical practice. Epidemiologic surveys have revealed that in the general population the median prevalence rate for any PD is about 10% (2); in specialized psychiatric care systems, prevalence estimates rapidly rise to ≥50% (3, 4). However, inspection of official clinical records of in- and outpatient services would provide a completely different picture, because PDs are markedly underdiagnosed by clinicians (5, 6). As a matter of fact, the diagnosis seldom appears in official clinical records. Tyrer et al. (1) suggest that less than 5% of all hospital admissions are officially recorded with a PD diagnosis. This implies that most patients with severe personality pathology are primarily diagnosed with and treated for other, often secondary and subsequent, mental disorders. Therefore, this review emphasizes ways in which maladaptive and pathological personality challenges routine psychiatric practice and why specific consideration of personality is warranted for the global provision and distribution of mental health services.

A comprehensive review of the adverse impact of personality on psychosocial functioning and mental health stringently needs to incorporate normal personality traits such as the Big Five, which comprise neuroticism, extraversion, agreeableness, conscientiousness, and openness (7). The categorical PD conceptualization included in DSM-5 (8) and ICD-10 (9) lacks accuracy and adequacy, and there is clear evidence favoring a dimensional PD conceptualization over the existing system with its arbitrary categories (10–12). In the year 2007, in view of the upcoming DSM-5, the majority of PD experts, comprising clinicians and researchers, agreed that PDs are best viewed as personality dimensions and that the categorical system incorporated in DSM-5 and ICD-10 should be replaced (13). In support of this view, findings from original studies (14, 15), meta-analyses (16, 17), and comprehensive reviews (18, 19) consistently demonstrate that normal and pathological personality are different manifestations of the same underlying latent spectrum of general personality functioning. In particular, neuroticism closely relates to general personality dysfunction and shows substantial overlap with most PD diagnoses (14, 17, 20). Since, a detailed account of the dimensional structure of normal and pathological personality is beyond the scope of this paper, interested readers are referred to Widiger and Simonsen (19).

In order to draw a comprehensive picture of the relevance of personality for public mental health and psychiatric practice, a thorough evaluation of findings from personality psychology research is necessary, adding valuable information to the traditional psychiatric research on PD diagnoses. This is particularly true since PD diagnoses and pathological personality traits are best viewed as extreme variants on general personality domains. Thus, in this narrative review, I will outline the empirical research literature on the pervasive impact of both normal and pathological personality. My main objective is to provide a comprehensive review of the literature that is aimed at demonstrating why a thorough assessment of personality is indispensable for psychiatric practice. In order to cover a broad range of public mental health issues, I will focus on the following four major targets of psychiatric practice: first, social functioning; second, occurrence of common mental disorders; third, course and remission of psychopathological syndromes; and fourth, service use and treatment response. This review will not deal with neurophysiological and endocrine pathways that may account for the association between personality and mental health. Such a discussion is beyond the scope of this paper and is better suited to other specialties. Readers interested in the biological bases of personality are for instance referred to the review by Depue and Fu (21).

## Impact of Personality on Social Functioning

Personality has a significant impact on almost all areas of human life (22). By implication, this review can only focus on a few aspects that I have chosen for their face validity and their implications for psychiatric practice. My review of the impact on social functioning will thus mainly touch on aspects of interpersonal and occupational functioning. Both of these topics are known to influence public mental health and are of considerable relevance for mental health policy and psychiatric practice (23–25).

First, with respect to interpersonal functioning, it has consistently been shown that normal personality traits substantially relate to relational ruptures, interpersonal conflicts, and separation or divorce. For instance, using data from a prospective longitudinal study, Donnellan et al. (26) demonstrated that neuroticism in particular had a significant negative influence on subsequent relationship quality. Jockin et al. (27), using a genetic analysis of an adult twin sample, estimated that in women and men a remarkable proportion of 30 and 42%, respectively, of the heritability of the genetic vulnerability for divorce was accounted for by personality. A meta-analysis of longitudinal studies confirmed that personality traits – specifically high neuroticism, low conscientiousness and low agreeableness – substantially predict divorce (28). Moreover, several large epidemiological studies have shown that general personality dysfunction and PD diagnoses relate to social dysfunction, interpersonal conflicts, and separation or divorce (29–31).

Another consistently replicated epidemiologic finding is the association of personality pathology with low educational achievement, low income, and unemployment (29, 32). Hengartner et al. (33) showed that PD traits significantly relate to various adverse occupational outcomes, such as severe conflicts in the workplace and dismissal or demotion. Correspondingly, there is ample evidence that PDs strongly increase individuals’ risk for disability pensioning (34). Research in personnel and organizational psychology supports these findings. For instance, Wille et al. (35) showed in a prospective longitudinal study over 15 years that maladaptive personality traits negatively relate to desirable work outcomes such as career and job satisfaction, whereas they positively predict adverse outcomes such as job stress. In a meta-analysis of occupational performance motivation, Judge and Ilies (36) confirmed the substantial association between personality and performance motivation as expressed by effect sizes of *r* = −0.31 for neuroticism and *r* = 0.24 for conscientiousness. In another meta-analysis, Salgado (37) likewise demonstrated that neuroticism and conscientiousness were valid predictors for job performance across various job criteria and occupational groups. Finally, using data from the Netherlands Mental Health Survey and Incidence Study (NEMESIS), Michon et al. (38) showed that in persons with common mental disorders, baseline personality traits fully account for subsequent work impairment.

In conclusion, the studies outlined above emphasize the predominant role that personality plays as an independent risk factor for global functional impairment. A stable and supportive romantic relationship, a regular income, and a fulfilling job are important resources for psychiatric patients. Since maladaptive personality compromises these domains of social functioning, it poses a serious threat to psychiatric practice. Clinicians should thus be aware that maladaptive personality significantly impairs their patients’ social functioning and that high scores on specific personality traits undermine powerful resources, which in turn has a negative impact on therapeutic progress and patients’ wellbeing.

## Impact of Personality on Incidence and Prevalence of Common Mental Disorders

Research on both normal and pathological personality has stressed the strong and consistent association between personality and the occurrence of mental disorders (22, 39, 40). There is compelling evidence from two meta-analyses that specifically neuroticism and to a lesser extent also low conscientiousness (i.e., disorderliness and impulsivity) substantially relate to mood, anxiety, and substance use disorders. Low agreeableness (i.e., antagonism and aggressiveness) is associated with externalizing disorders and introversion specifically with internalizing disorders (41, 42). Moreover, neuroticism constitutes a broad vulnerability factor for the co-occurrence within and between both internalizing and externalizing disorders (43, 44). Thus, in sum, cross-sectional epidemiological studies provide compelling evidence that neuroticism in particular is strongly associated with the occurrence and co-occurrence of all common mental disorders as expressed by large effect sizes of *d* > 0.8 or *r* > 0.5. Neuroticism is also the most important trait underlying general personality dysfunction and specific PD diagnoses (14, 17, 20). It consistently follows that the severity of personality pathology as well as PD diagnoses substantially relate to co-occurring mood, anxiety, and substance use disorders (32, 45) and to the number of co-occurring mental disorders (46, 47). However, correlation does not imply causation, which is why cross-sectional studies are of limited validity for aetiopathological models. Only controlled longitudinal designs provide predictive validity for a construct and allow drawing stringent causal conclusions.

The few longitudinal surveys that included PDs produced consistent results that corroborate the status of PDs as crucial risk factors for the onset of mental disorders. Using data from the Baltimore Epidemiologic Catchment Area (ECA) study, Bienvenu et al. (48) showed that baseline PD traits significantly predicted first-onset panic disorder and agoraphobia over the follow-up period. The Children in the Community Study revealed that PDs in adolescence significantly increase the risk for anxiety disorders, mood disorders, substance use disorders, ADHD and other disruptive disorders, and various educational and social problems in adulthood (49, 50). Finally, using data from the first and second waves of the National Epidemiologic Survey on Alcohol and Related Conditions (NESARC), Grant et al. (51) likewise found that baseline PDs predicted the subsequent 12-month incidence of mood, anxiety, and substance use disorders.

Compelling evidence for a causal link also comes from normal personality research. In longitudinal surveys, neuroticism in particular demonstrated substantial predictive validity for the occurrence of mental disorders [for a comprehensive review on neuroticism, see Lahey (52)]. In more detail, Kendler et al. (53) showed that neuroticism strongly predicts the risk for lifetime and new-onset major depression and that neuroticism considerably reflects the genetic liability to depression. In other studies, Kendler and colleagues consolidated the association between neuroticism and depression by reporting that neuroticism moderates the impact of adverse life events on major depression (54) and by demonstrating that the genetic liability to depression alters people’s sensitivity to adverse life events (55, 56). Moreover, longitudinal data from the Christchurch Health and Development Study (57) as well as from a prospective longitudinal clinical study with adolescent inpatients (58) showed that neuroticism prospectively relates to suicidal ideation and suicide attempts.

It is important to note that neuroticism by no means exclusively relates to conceptually overlapping constructs such as depressiveness or anxiousness, which are *per se* specific facets of neuroticism. Linking neuroticism exclusively to symptoms of negative affectivity might thus appear circular or redundant. However, the predictive validity of neuroticism is not at all restricted to affective disorders. For instance, Van Os and Jones (59) showed in a large birth cohort that neuroticism at age 16 increases the risk, whereas extraversion reduces the risk for subsequent schizophrenia in adult life. Data from the Prospective Zurich Cohort Study revealed that variance in the expression of subclinical psychosis symptoms as repeatedly assessed from age 20 to 50 years is predominantly caused by stable traits (60). Moreover, the facets of neuroticism, here especially depressiveness, substantially relate to the latent trait underlying the occurrence of subclinical psychosis (60). In another analysis of this prospectively followed cohort, Leeners et al. (61) found that in women the baseline personality facets of nervousness, aggressiveness, depressiveness, irritability, and openness increase the risk, whereas sociability reduces the risk for subsequent sexual difficulties with reaching orgasm. Finally, Turiano et al. (62), using data from the Midlife Development in the United States (MIDUS) survey, showed that increases in neuroticism and openness predict progressive substance use, while increases in conscientiousness and agreeableness predict declines in substance use over time. In addition, in that particular study, conscientiousness was an important moderator of the effects that personality traits have on substance use (62).

Thus, taken together, these findings clearly demonstrate that persons with maladaptive personality traits are at highly increased risk for the development of subsequent mental disorders and other psychological difficulties. As a consequence, these at-risk patients should be observed and followed carefully once they have entered the health care system. Prerequisite to this recommendation is of course a thorough assessment of personality in every single patient as early as possible in the clinical evaluation process.

## Impact of Personality on Course and Remission of Psychopathological Syndromes

Focusing exclusively on the occurrence of mental disorders in the general population would draw an incomplete picture of the pervasive impact of personality. The effect of personality on the course and persistence of already existing mental disorders, that is, the primary disorders for which persons are referred to mental health services, is presumably of even greater relevance for clinicians’ primary considerations in routine practice. Since most clinicians principally record and treat mental disorders, but not underlying pathological personality traits, we deliberately focus on the literature on common mental disorders and not on the course and stability of PDs as primary targets of intervention. Readers interested in the treatment and course of PDs may consult reviews by Bateman et al. (63) and Newton-Howes et al. (64).

Moran et al. (65) demonstrated in a 2-year longitudinal follow-up study of patients with the primary diagnosis of psychosis that independent of other baseline covariates, comorbid PD increased the odds of attempted or completed suicide over the observation period by 87%. Data from the NESARC revealed that in the general population the prevalence of a PD diagnosis, in particular, antisocial, borderline, and schizotypal PD, significantly increases the risk of persistent and addictive drug use (66), which conforms with the impact of high neuroticism and low conscientiousness on substance use as detailed above [see Ref. (62)]. A 10-year longitudinal study of psychiatric patients with major depression and/or dysthymic disorder demonstrated that among various baseline characteristics, Cluster B PD (predominantly depicting the domain of negative affectivity) was the only robust and independent predictor of suicide attempts at follow-up (67). Massion et al. (68) showed that in patients with generalized anxiety disorder and social phobia, baseline PDs reduced remission rates by 30 and 39%, respectively, over a 5-year follow-up period. In another prospective, longitudinal study of patients with affective disorders, baseline severity of personality pathology significantly predicted persistent impairment in the social functioning of those patients over the 12-year observation period, even when baseline psychopathology was adjusted for (69). Using the same data, Tyrer et al. (70) additionally found that baseline personality pathology significantly impeded the remission of anxiety symptoms at 12-year follow-up. Accordingly, the authors concluded that PDs may predispose to treatment resistance and chronicity of affective disorders (70).

Thus, as stated in the preceding section, a well-conceived treatment planning for common mental disorders stringently needs to incorporate maladaptive personality traits. Only when personality has been taken into account and treated in a timely fashion (that is, as early as possible), can clinicians possibly prevent persistent drug use, long-term dysfunction, and a chronic course of illness. The evidence presented here clearly shows that patients with personality pathology have more severe, persistent, and recurring mental disorders than do patients without personality pathology. It is therefore crucial to consider the impact of personality right at the outset of clinical evaluations when different treatments are gauged (for instance, whether a patient should receive intensive case management or not).

## Impact of Personality on Service Use and Treatment Response

In contrast to the findings related to aspects of course and persistency of psychopathological syndromes outlined above, in this section I will introduce studies that provide evidence for the influence of personality specifically on service use and treatment response. To begin with, it is important to stress that personality significantly interferes with health care utilization, which poses a serious issue for health economics and resources in mental health practice. For instance, using data from the MIDUS survey, a large epidemiological study demonstrated that in the general population neuroticism in particular relates to the increased likelihood of mental health service use (71). Findings from the NEMESIS confirmed the crucial role of neuroticism by demonstrating that this particular personality trait increases the use of both primary and specialized mental health care (72). In addition, in that same study, it was also shown that once entered into the mental health care system, patients scoring high on neuroticism make more repeated visits. The authors argued that persons scoring high on negative affectivity (typically borderline patients) are vulnerable to stress and lack appropriate coping strategies, which is why they need intensive professional help (72). Those conclusions conform perfectly with the findings by Kendler et al. (54, 56) detailed above on the interrelationship between neuroticism, stressful life events, and the occurrence of depression.

Finally, personality not only influences service utilization but also the efficacy of and compliance with mental health treatments. For instance, a large longitudinal clinical study with over 600 patients with major depressive disorder revealed that low neuroticism and high extraversion and openness predict response to both pharmacotherapy and psychotherapy (73). Addressing a similar aim but using a completely different setting, which compared group vs. internet-based cognitive behavior therapy, Spek et al. (74) found that lower baseline neuroticism significantly predicts better outcomes in both treatments. Based on a comprehensive literature review, Mulder (75) noted that personality, particularly neuroticism, generally predicts worse treatment outcomes, but that this association is not unequivocally clear and apparently depends on the study design. In contrast to that rather cautious verdict, a meta-analysis of the effect of PDs on treatment outcome in depression corroborates the detrimental impact of maladaptive personality traits (76). The robust result of this study revealed that concurrent PD doubles the risk for a poor treatment outcome in major depression across various treatments (pharmacological and psychological alone, or combined).

Newton-Howes et al. (76) conclude that “a diagnosis of personality disorder is not necessarily a poor prognostic indicator. These patients simply require treatment of both the personality disorder and the depression. This offers a challenge to clinicians. Despite our best endeavors patients with personality disorder remain one of the most difficult groups in psychiatric practice (p. 18)”. There is not much to add to this concise statement except to reiterate that clinicians can avoid treatment resistance and poor outcomes only if, first, they are fully aware of their patients’ underlying personality pathology, and, second, if personality is stringently included in the treatment plan. Moreover, clinicians need to consider that patients scoring high on the personality trait of negative affectivity (that is, excessive neuroticism and respective Cluster B and C PDs) lack adequate coping resources. These patients are thus highly vulnerable to environmental stressors and negative life events, which is why they need ongoing long-term treatment and thorough supervision.

## Conclusion

The eminent studies summarized in this narrative review provide compelling evidence for the pervasive and persistent effect of maladaptive personality, in particular negative affectivity (i.e., excessively high neuroticism) and the severity of general personality dysfunction (as reflected by the diagnosis of one or more PDs), on a wide variety of clinically relevant adverse outcomes. Several renowned PD experts with profound knowledge of the scientific literature and with extensive experience in clinical practice, including Tyrer et al. (1) and Krueger and Eaton (40), suggest that a thorough examination of personality should be a mandatory and integral part of clinical assessment, prognosis, and treatment planning. However, any reader with clinical experience will, unfortunately, have to admit that this suggestion is far from being followed in routine psychiatric practice. Too many mental health professionals still neglect the pervasive impact of overt personality pathology, and many professionals are even less aware of the covert latent personality traits that underlie manifest psychopathological syndromes. In this respect, I hope that this review helps to give maladaptive personality traits the clinical attention that they deserve.

Tyrer (77) posits that pathological personality is the cause of all severe forms of persistent and recurrent non-cognitive mental disorders. This narrative review, although far from being conclusive, provides compelling evidence in support of this hypothesis. The implications for psychiatric practice provided at the end of each respective section deliberately remind the reader of two major points. First, maladaptive personality, in particular the spectrum of negative affectivity, substantially increases the risk of severe psychopathological syndromes, and pervasively impairs functioning, treatment response, symptom remission, and recovery. Second, clinicians should adopt routine assessment of their patients’ personality as early as possible in the clinical process and incorporate this important information in their treatment decisions. Having this said, it should also be acknowledged that the assessment of maladaptive personality and the diagnosis of PDs are not that straightforward as this review might suggest. In fact, the assessment of PDs poses a challenge to psychiatric practice on its own, because there is no accepted gold standard and each assessment method has its limitations (78). These difficulties are not only due to the inadequate classification of maladaptive personality in DSM-5 and ICD-10 but also caused by the very intricate nature of personality traits and personality functioning (79). Research in normal and pathological personality has demonstrated that the accordance between self- and informant-reports is rather modest (80, 81), although both sources have considerable predictive validity and both provide unique information that is important to the understanding of personality traits and PDs (10, 79–81). The overlap between personality and mental disorders and the impact of acute psychopathological symptoms on the assessment of personality make this demanding task even more difficult. Therefore, the general consensus is that a multiple-informant assessment over multiple time points is the most accurate method for both the assessment of personality traits and the diagnosis of PDs. For a thorough discussion of these methodological issues, the interested reader is referred to the literature.

Finally, although not the primary aim of this review, I would like to suggest that researchers should at least consider including a short personality assessment in their study designs. By doing so, they may come to see that personality independently accounts for many important associations in mental health research, even in domains where it was not expected.

## Conflict of Interest Statement

The author declares that the research was conducted in the absence of any commercial or financial relationships that could be construed as a potential conflict of interest.

## References

[1] Tyrer P Reed GM Crawford MJ. Classification, assessment, prevalence, and effect of personality disorder. Lancet (2015) 385(9969):717–26. 10.1016/S0140-6736(14)61995-4 25706217

[2] Samuels J. Personality disorders: epidemiology and public health issues. Int Rev Psychiatry (2011) 23(3):223–33. 10.3109/09540261.2011.588200 21923224

[3] Beckwith H Moran PF Reilly J. Personality disorder prevalence in psychiatric outpatients: a systematic literature review. Personal Ment Health (2014) 8(2):91–101. 10.1002/pmh.1252 24431304

[4] Zimmerman M Chelminski I Young D. The frequency of personality disorders in psychiatric patients. Psychiatr Clin North Am (2008) 31(3):405–20. 10.1016/j.psc.2008.03.015 18638643

[5] Oldham JM Skodol AE Personality disorders in the public sector. Hosp Community Psychiatry (1991) 42(5):481–7. 2060913 10.1176/ps.42.5.481

[6] Zimmerman M Rothschild L Chelminski I. The prevalence of DSM-IV personality disorders in psychiatric outpatients. Am J Psychiatry (2005) 162(10):1911–8. 10.1176/appi.ajp.162.10.1911 16199838

[7] Goldberg LR The structure of phenotypic personality-traits. Am Psychol (1993) 48(1):26–34. 10.1037/0003-066X.48.1.26 8427480

[8] American Psychiatric Association. Diagnostic and Statistical Manual of Mental Disorders DSM-5. Washington, DC: American Psychiatric Association (2013).

[9] World Health Organization. International Classification of Diseases ICD-10. 10th ed Geneva: World Health Organization (1992).

[10] Clark LA. Assessment and diagnosis of personality disorder: perennial issues and an emerging reconceptualization. Annu Rev Psychol (2007) 58:227–57. 10.1146/annurev.psych.57.102904.190200 16903806

[11] Farmer RF. Issues in the assessment and conceptualization of personality disorders. Clin Psychol Rev (2000) 20(7):823–51. 10.1016/S0272-7358(99)00014-8 11057374

[12] Trull TJ Durrett CA. Categorical and dimensional models of personality disorder. Annu Rev Clin Psychol (2005) 1:355–80. 10.1146/annurev.clinpsy.1.102803.144009 17716092

[13] Bernstein DP Iscan C Maser J. Boards of directors of the association for research in personality D, international society for the study of personality D. Opinions of personality disorder experts regarding the DSM-IV personality disorders classification system. J Pers Disord (2007) 21(5):536–51. 10.1521/pedi.2007.21.5.536 17953505

[14] Hengartner MP Ajdacic-Gross V Rodgers S Müller M Rössler W. The joint structure of normal and pathological personality: further evidence for a dimensional model. Compr Psychiatry (2014) 55(3):667–74. 10.1016/j.comppsych.2013.10.011 24314825

[15] Thomas KM Yalch MM Krueger RF Wright AG Markon KE Hopwood CJ. The convergent structure of DSM-5 personality trait facets and five-factor model trait domains. Assessment (2013) 20(3):308–11. 10.1177/1073191112457589 22946103

[16] Markon KE Krueger RF Watson D. Delineating the structure of normal and abnormal personality: an integrative hierarchical approach. J Pers Soc Psychol (2005) 88(1):139–57. 10.1037/0022-3514.88.1.139 15631580 PMC2242353

[17] Samuel DB Widiger TA. A meta-analytic review of the relationships between the five-factor model and DSM-IV-TR personality disorders: a facet level analysis. Clin Psychol Rev (2008) 28(8):1326–42. 10.1016/j.cpr.2008.07.002 18708274 PMC2614445

[18] Widiger TA Livesley WJ Clark LA. An integrative dimensional classification of personality disorder. Psychol Assess (2009) 21(3):243–55. 10.1037/a0016606 19719338

[19] Widiger TA Simonsen E. Alternative dimensional models of personality disorder: finding a common ground. J Pers Disord (2005) 19(2):110–30. 10.1521/pedi.19.2.110.62628 15899712

[20] Kendler KS Aggen SH Czajkowski N Roysamb E Tambs K Torgersen S The structure of genetic and environmental risk factors for DSM-IV personality disorders: a multivariate twin study. Arch Gen Psychiatry (2008) 65(12):1438–46. 10.1001/archpsyc.65.12.1438 19047531 PMC2844885

[21] Depue RA Fu Y. Neurogenetic and experiential processes underlying major personality traits: implications for modelling personality disorders. Int Rev Psychiatry (2011) 23(3):258–81. 10.3109/09540261.2011.599315 21923227

[22] Ozer DJ Benet-Martinez V. Personality and the prediction of consequential outcomes. Annu Rev Psychol (2006) 57:401–21. 10.1146/annurev.psych.57.102904.190127 16318601

[23] O’Brien A Fahmy R Singh SP. Disengagement from mental health services. A literature review. Soc Psychiatry Psychiatr Epidemiol (2009) 44(7):558–68. 10.1007/s00127-008-0476-0 19037573

[24] Pevalin DJ Goldberg DP. Social precursors to onset and recovery from episodes of common mental illness. Psychol Med (2003) 33(2):299–306. 10.1017/S0033291702006864 12622308

[25] Wang PS Lane M Olfson M Pincus HA Wells KB Kessler RC. Twelve-month use of mental health services in the United States: results from the National Comorbidity Survey Replication. Arch Gen Psychiatry (2005) 62(6):629–40. 10.1001/archpsyc.62.6.629 15939840

[26] Donnellan MB Larsen-Rife D Conger RD. Personality, family history, and competence in early adult romantic relationships. J Pers Soc Psychol (2005) 88(3):562–76. 10.1037/0022-3514.88.3.562 15740446

[27] Jockin V McGue M Lykken DT. Personality and divorce: a genetic analysis. J Pers Soc Psychol (1996) 71(2):288–99. 10.1037/0022-3514.71.2.288 8765483

[28] Roberts BW Kuncel NR Shiner R Caspi A Goldberg LR The power of personality the comparative validity of personality traits, socioeconomic status, and cognitive ability for predicting important life outcomes. Perspect Psychol Sci (2007) 2(4):313–45. 10.1111/j.1745-6916.2007.00047.x PMC4499872 26151971

[29] Coid J Yang M Tyrer P Roberts A Ullrich S. Prevalence and correlates of personality disorder in Great Britain. Br J Psychiatry (2006) 188:423–31. 10.1192/bjp.188.5.423 16648528

[30] Hengartner MP Müller M Rodgers S Rössler W Ajdacic-Gross V. Interpersonal functioning deficits in association with DSM-IV personality disorder dimensions. Soc Psychiatry Psychiatr Epidemiol (2014) 49(2):317–25. 10.1007/s00127-013-0707-x 23674198

[31] Hopwood CJ Malone JC Ansell EB Sanislow CA Grilo CM McGlashan TH Personality assessment in DSM-5: empirical support for rating severity, style, and traits. J Pers Disord (2011) 25(3):305–20. 10.1521/pedi.2011.25.3.305 21699393

[32] Yang M Coid J Tyrer P. Personality pathology recorded by severity: national survey. Br J Psychiatry (2010) 197(3):193–9. 10.1192/bjp.bp.110.078956 20807963

[33] Hengartner MP Müller M Rodgers S Rössler W Ajdacic-Gross V. Occupational functioning and work impairment in association with personality disorder trait-scores. Soc Psychiatry Psychiatr Epidemiol (2014) 49(2):327–35. 10.1007/s00127-013-0739-2 23835577

[34] Ostby KA Czajkowski N Knudsen GP Ystrom E Gjerde LC Kendler KS Personality disorders are important risk factors for disability pensioning. Soc Psychiatry Psychiatr Epidemiol (2014) 49(12):2003–11. 10.1007/s00127-014-0878-0 24791656 PMC4218874

[35] Wille B De Fruyt F De Clercq B Expanding and reconceptualizing aberrant personality at work: validity of five-factor model aberrant personality tendencies to predict career outcomes. Pers Psychol (2013) 66(1):173–223. 10.1111/Peps.12016

[36] Judge TA Ilies R. Relationship of personality to performance motivation: a meta-analytic review. J Appl Psychol (2002) 87(4):797–807. 12184582 10.1037/0021-9010.87.4.797

[37] Salgado JF. The five factor model of personality and job performance in the european community. J Appl Psychol (1997) 82(1):30–43. 10.1037/0021-9010.82.1.30 9119797

[38] Michon HW ten Have M Kroon H van Weeghel J de Graaf R Schene AH. Mental disorders and personality traits as determinants of impaired work functioning. Psychol Med (2008) 38(11):1627–37. 10.1017/S0033291707002449 18205968

[39] Clark LA. Temperament as a unifying basis for personality and psychopathology. J Abnorm Psychol (2005) 114(4):505–21. 10.1037/0021-843X.114.4.505 16351374

[40] Krueger RF Eaton NR Personality traits and the classification of mental disorders: toward a more complete integration in DSM-5 and an empirical model of psychopathology. Personal Disord (2010) 1(2):97–118. 10.1037/a0018990 22448621

[41] Kotov R Gamez W Schmidt F Watson D. Linking “big” personality traits to anxiety, depressive, and substance use disorders: a meta-analysis. Psychol Bull (2010) 136(5):768–821. 10.1037/A0020327 20804236

[42] Malouff JM Thorsteinsson EB Schutte NS The relationship between the five-factor model of personality and symptoms of clinical disorders: a meta-analysis. J Psychopathol Behav Assess (2005) 27(2):101–14. 10.1007/s10862-005-5384-y

[43] Khan AA Jacobson KC Gardner CO Prescott CA Kendler KS Personality and comorbidity of common psychiatric disorders. Br J Psychiatry (2005) 186:190–6. 10.1192/bjp.186.3.190 15738498

[44] Weinstock LM Whisman MA. Neuroticism as a common feature of the depressive and anxiety disorders: a test of the revised integrative hierarchical model in a national sample. J Abnorm Psychol (2006) 115(1):68–74. 10.1037/0021-843X.115.1.68 16492097

[45] Jackson HJ Burgess PM. Personality disorders in the community: a report from the Australian National Survey of Mental Health and Wellbeing. Soc Psychiatry Psychiatr Epidemiol (2000) 35:531–8. 10.1007/s001270050276 11213842

[46] Hengartner MP De Fruyt F Rodgers S Müller M Rössler W Ajdacic-Gross V. An integrative examination of general personality dysfunction in a large community sample. Personal Ment Health (2014) 8(4):276–89. 10.1002/pmh.1263 25044701

[47] Lenzenweger MF Lane MC Loranger AW Kessler RC. DSM-IV personality disorders in the National Comorbidity Survey Replication. Biol Psychiatry (2007) 62(6):553–64. 10.1016/j.biopsych.2006.09.019 17217923 PMC2044500

[48] Bienvenu OJ Stein MB Samuels JF Onyike CU Eaton WW Nestadt G. Personality disorder traits as predictors of subsequent first-onset panic disorder or agoraphobia. Compr Psychiatry (2009) 50(3):209–14. 10.1016/j.comppsych.2008.08.006 19374963 PMC2691589

[49] Johnson JG Cohen P Skodol AE Oldham JM Kasen S Brook JS. Personality disorders in adolescence and risk of major mental disorders and suicidality during adulthood. Arch Gen Psychiatry (1999) 56(9):805–11. 10.1001/archpsyc.56.9.805 12884886

[50] Johnson JG First MB Cohen P Skodol AE Kasen S Brook JS. Adverse outcomes associated with personality disorder not otherwise specified in a community sample. Am J Psychiatry (2005) 162(10):1926–32. 10.1176/appi.ajp.162.10.1926 16199840

[51] Grant BF Goldstein RB Chou SP Huang B Stinson FS Dawson DA Sociodemographic and psychopathologic predictors of first incidence of DSM-IV substance use, mood and anxiety disorders: results from the Wave 2 National Epidemiologic Survey on Alcohol and Related Conditions. Mol Psychiatry (2009) 14(11):1051–66. 10.1038/mp.2008.41 18427559 PMC2766434

[52] Lahey BB. Public health significance of neuroticism. Am Psychol (2009) 64(4):241–56. 10.1037/a0015309 19449983 PMC2792076

[53] Kendler KS Gatz M Gardner CO Pedersen NL. Personality and major depression: a Swedish longitudinal, population-based twin study. Arch Gen Psychiatry (2006) 63(10):1113–20. 10.1001/archpsyc.63.10.1113 17015813

[54] Kendler KS Kuhn J Prescott CA. The interrelationship of neuroticism, sex, and stressful life events in the prediction of episodes of major depression. Am J Psychiatry (2004) 161(4):631–6. 10.1176/appi.ajp.161.4.631 15056508

[55] Kendler KS Kessler RC Walters EE MacLean C Neale MC Heath AC Stressful life events, genetic liability, and onset of an episode of major depression in women. Am J Psychiatry (1995) 152(6):833–42. 10.1176/ajp.152.6.833 7755111

[56] Kendler KS Kuhn JW Vittum J Prescott CA Riley B. The interaction of stressful life events and a serotonin transporter polymorphism in the prediction of episodes of major depression: a replication. Arch Gen Psychiatry (2005) 62(5):529–35. 10.1001/archpsyc.62.5.529 15867106

[57] Fergusson DM Woodward LJ Horwood LJ. Risk factors and life processes associated with the onset of suicidal behaviour during adolescence and early adulthood. Psychol Med (2000) 30(1):23–39. 10.1017/S003329179900135X 10722173

[58] Enns MW Cox BJ Inayatulla M. Personality predictors of outcome for adolescents hospitalized for suicidal ideation. J Am Acad Child Adolesc Psychiatry (2003) 42(6):720–7. 10.1097/01.CHI.0000046847.56865.B0 12921480

[59] Van Os J Jones PB. Neuroticism as a risk factor for schizophrenia. Psychol Med (2001) 31(6):1129–34. 10.1017/S0033291701004044 11513380

[60] Rössler W Hengartner MP Ajdacic-Gross V Haker H Angst J. Deconstructing sub-clinical psychosis into latent-state and trait variables over a 30-year time span. Schizophr Res (2013) 150(1):197–204. 10.1016/j.schres.2013.07.042 23932663

[61] Leeners B Hengartner MP Rössler W Ajdacic-Gross V Angst J. The role of psychopathological and personality covariates in orgasmic difficulties: a prospective longitudinal evaluation in a cohort of women from age 30 to 50. J Sex Med (2014) 11(12):2928–37. 10.1111/jsm.12709 25358802

[62] Turiano NA Whiteman SD Hampson SE Roberts BW Mroczek DK. Personality and substance use in midlife: conscientiousness as a moderator and the effects of trait change. J Res Pers (2012) 46(3):295–305. 10.1016/j.jrp.2012.02.009 22773867 PMC3388488

[63] Bateman AW Gunderson J Mulder R Treatment of personality disorder. Lancet (2015) 385(9969):735–43. 10.1016/S0140-6736(14)61394-5 25706219

[64] Newton-Howes G Clark LA Chanen A Personality disorder across the life course. Lancet (2015) 385(9969):727–34. 10.1016/S0140-6736(14)61283-6 25706218

[65] Moran P Walsh E Tyrer P Burns T Creed F Fahy T. Does co-morbid personality disorder increase the risk of suicidal behaviour in psychosis? Acta Psychiatr Scand (2003) 107(6):441–8. 12752021 10.1034/j.1600-0447.2003.00125.x

[66] Fenton MC Keyes K Geier T Greenstein E Skodol A Krueger B Psychiatric comorbidity and the persistence of drug use disorders in the United States. Addiction (2012) 107(3):599–609. 10.1111/j.1360-0443.2011.03638.x 21883607 PMC3260401

[67] May AM Klonsky ED Klein DN. Predicting future suicide attempts among depressed suicide ideators: a 10-year longitudinal study. J Psychiatr Res (2012) 46(7):946–52. 10.1016/j.jpsychires.2012.04.009 22575331 PMC3372684

[68] Massion AO Dyck IR Shea MT Phillips KA Warshaw MG Keller MB. Personality disorders and time to remission in generalized anxiety disorder, social phobia, and panic disorder. Arch Gen Psychiatry (2002) 59(5):434–40. 10.1001/archpsyc.59.5.434 11982447

[69] Seivewright H Tyrer P Johnson T. Persistent social dysfunction in anxious and depressed patients with personality disorder. Acta Psychiatr Scand (2004) 109(2):104–9. 10.1046/j.1600-0447.2003.00241.x 14725590

[70] Tyrer P Seivewright H Johnson T. The Nottingham study of neurotic disorder: predictors of 12-year outcome of dysthymic, panic and generalized anxiety disorder. Psychol Med (2004) 34(8):1385–94. 15724870 10.1017/s0033291704002569

[71] Goodwin RD Hoven CW Lyons JS Stein MB. Mental health service utilization in the United States. The role of personality factors. Soc Psychiatry Psychiatr Epidemiol (2002) 37(12):561–6. 12545232 10.1007/s00127-002-0563-6

[72] ten Have M Oldehinkel A Vollebergh W Ormel J. Does neuroticism explain variations in care service use for mental health problems in the general population? Results from the Netherlands mental health survey and incidence study (NEMESIS). Soc Psychiatry Psychiatr Epidemiol (2005) 40(6):425–31. 10.1007/s00127-005-0916-z 16003591

[73] Quilty LC De Fruyt F Rolland JP Kennedy SH Rouillon PF Bagby RM. Dimensional personality traits and treatment outcome in patients with major depressive disorder. J Affect Disord (2008) 108(3):241–50. 10.1016/j.jad.2007.10.022 18067975

[74] Spek V Nyklicek I Cuijpers P Pop V. Predictors of outcome of group and internet-based cognitive behavior therapy. J Affect Disord (2008) 105(1–3):137–45. 10.1016/j.jad.2007.05.001 17543392

[75] Mulder RT Personality pathology and treatment outcome in major depression: a review. Am J Psychiatry (2002) 159(3):359–71. 11869996 10.1176/appi.ajp.159.3.359

[76] Newton-Howes G Tyrer P Johnson T. Personality disorder and the outcome of depression: meta-analysis of published studies. Br J Psychiatry (2006) 188:13–20. 10.1192/bjp.188.1.13 16388064

[77] Tyrer P Personality dysfunction is the cause of recurrent non-cognitive mental disorder: a testable hypothesis. Personal Ment Health (2015) 9(1):1–7. 10.1002/pmh.1255 24599840

[78] Zimmerman M. Diagnosing personality disorders: a review of issues and research methods. Arch Gen Psychiatry (1994) 51(3):225–45. 10.1001/archpsyc.1994.03950030061006 8122959

[79] Tyrer P Coombs N Ibrahimi F Mathilakath A Bajaj P Ranger M Critical developments in the assessment of personality disorder. Br J Psychiatry Suppl (2007) 49:s51–9. 10.1192/bjp.190.5.s51 17470943

[80] Klonsky ED Oltmanns TF Turkheimer E Informant-reports of personality disorder: relation to self-reports and future research directions. Clin Psychol Sci Pract (2002) 9:300–11. 10.1093/clipsy.9.3.300

[81] Meyer GJ Finn SE Eyde LD Kay GG Moreland KL Dies RR Psychological testing and psychological assessment – a review of evidence and issues. Am Psychol (2001) 56(2):128–65. 10.1037/0003-066X.56.2.128 11279806

